# ERCC1 which affects lipids metabolism and actin dynamics in coal workers’ pneumoconiosis is a candidate biomarker for early warning and diagnosis

**DOI:** 10.1371/journal.pone.0308082

**Published:** 2024-09-16

**Authors:** Hao Deng, Yan Chen, Mali Wu, Tao Zhang

**Affiliations:** 1 Department of Occupational Diseases, Guiyang Public Health Clinical Center, Guiyang, GuiZhou, China; 2 Institute of Public Health, Guizhou CDC, Guiyang, GuiZhou, China; 3 College of Basic Medicine, Guizhou University of Traditional Chinese Medicine, Guiyang, GuiZhou, China; Cleveland Clinic Lerner Research Institute, UNITED STATES OF AMERICA

## Abstract

The single-nucleotide polymorphisms of genes related to DNA damage repair and inflammasomes and mutated gene expression in coal workers’ pneumoconiosis (CWP) were analysed to identify the risk factors of CWP and potential biomarkers for early warning and diagnosis. Further, mutated gene pathways were analysed based on proteome and metabolome. Han Chinese male subjects were randomly selected and divided into 4 or 5 groups according to the process of CWP. MassARRAY was used to sequence single-nucleotide polymorphism genotypes. Mutated gene expression in plasma was tested using enzyme-linked immunosorbent assay (ELISA). Odds ratios (ORs) and receiver operating characteristic curves (ROC) were calculated. The serum different proteins and metabolites were identified by Ultra Performance Liquid Chromatography Quadrupole time of flight/Mass Spectrum (UPLC-Q-TOF/MS) and analysed using bioinformation software. As CWP progressed, the CC and CA genotypes of *ERCC1* rs3212986 decreased and increased significantly, respectively. AA (OR = 3.016) and CA (OR = 2.130) genotypes were identified as risk factors for stage II. ERCC1 significantly decreased in processing of CWP. The cutoff value of ERCC1 was 5.265 pg/ml, with a sensitivity of 90.0% and specificity of 86.7%. ERCC1 had an indirect interaction with activator protein-1 and insulin and its pathways were mainly made with molecules related to lipid metabolism and actin dynamics. ERCC1 is a candidate biomarker for detection and precise intervention in CWP. If it reaches the threshold, workers will change other jobs in time and will not develop and diagnose as pneumoconiosis and will help the employers spend less money. Meanwhile, the signal molecules of ERCC1 pathway could be as a candidate target for drug discovery.

## Introduction

Coal workers’ pneumoconiosis (CWP) is a chronic occupational lung disease caused by the long-term inhalation of coal dust and primarily characterized by pulmonary inflammation and fibrosis progression [1]. China, as the world’s largest labour market with over 775 million workers, currently experiences the largest global health losses from pneumoconiosis [2]. By 2030, significantly reduce the number of deaths and illnesses caused by hazardous chemicals and air, water and soil pollution is as one of WHO SDG project [3]. The Chinese government has carried out the National Plan for the detection and Control of Occupational Diseases (2016–2020) to solve the current key and difficult problems in the detection and control of pneumoconiosis, resolutely curb the momentum of the high incidence of pneumoconiosis, and safeguard the rights and interests of workers’ occupational health, and has formulated the present action project [4]. As one of the most severe occupational diseases in China, CWP currently lacks specific and effective treatments, resulting in significant social and economic burden [5]. The clinical diagnosis of CWP mainly relies on occupational exposure history and abnormal imaging changes. However, as the disease is progressive and incurable, early prevention is particularly crucial [6]. But there are no specific indicators for early detection of pneumoconiosis, Imaging is not suitable for accurate early detection because it is highly subjective and with some lag in early detection. Therefore, obtaining effective early detection biomarkers for pneumoconiosis is important for early intervention of pneumoconiosis.

The pathogenesis of CWP is not fully understood. Inhaled dust particles activate pulmonary macrophages, which produce reactive oxygen species (ROS) and reactive nitrogen species, damaging pulmonary epithelial cells. ROS can also damage DNA, proteins, and cell membranes [7]. Conversely, ROS activate the immune system to produce an inflammatory response, with ROS and inflammation mutually promoting each other [8]. Therefore, the main characteristic of pulmonary fibrosis is persistent lung damage caused by oxidative stress and chronic inflammation [9]. Repeated stimulation of lung tissue by coal dust particles results in persistent inflammatory responses, leading to excessive collagen accumulation and diffuse fibrosis in the lungs [10]. High levels of DNA and chromosomal damage have been observed in coal miners, and patients with CWP are also susceptible to high levels of oxidative DNA damage caused by the long-term inhalation of dust particles [11–14]. Oxidative DNA damage may play a crucial role in the development and progression of CWP, and related DNA damage repair may be essential for determining the disease outcome.

The key molecule *ATM* rs189037 G/A, which is involved in repairing DNA double-strand breaks, is associated with increasing mutation rates and decreasing gene expression levels during the progression of CWP. It has been identified as a risk factor (GA odds ratio [OR] = 1.22 and AA OR = 1.12) [15], indicating that the ATM protein is suggested to play an important role in the progression of CWP. DNA damage repair in human cells includes six major pathways, including base excision repair (BER), nucleotide excision repair (NER), mismatch repair, homologous recombination, non-homologous end joining, and translesion synthesis [16]. The ERCC1-XPF heterodimer is an essential component of the NER pathway, which can catalyse the 5′ incision of the damaged DNA strand [17]. XRCC1 is a DNA repair scaffold protein that mainly supports BER and is responsible for correcting many minor base damages and single-strand breaks. BER is a highly coordinated process, and XRCC1 depletion can lead to loss of the core BER mechanism. Cells with XRCC1 defects exhibit reduced DNA repair, resulting in the accumulation of persistent DNA damage and increased genomic instability [18, 19]. However, it is unclear whether other single-nucleotide polymorphism (SNP) sites of ATM affect protein function in a similar manner to rs189037 G/A, and whether key molecules in the NER and BER pathways (ERCC1 and XRCC1) play similar roles in disease development to the ATM protein.

The intrinsic connection between oxidative stress response and inflammation suggests that molecules related to the inflammatory response may also have a significant impact on the development and progression of CWP. Inhibiting the inflammasome activation pathway or inflammasome-mediated cytokines can reduce fibrotic reactions in both in vitro and in vivo disease models [20]. Nucleotide-oligomerization domain-like receptor protein 1 (NLRP1), NLRP3, and NLRC4 are the most characteristic inflammasomes. Among them, the NLRP3 inflammasome plays an important role in silica-induced inflammation and fibrosis, and its inhibition results in the elimination of both inflammation and fibrosis [21]. Similar to NLRP3, NLRP1, when assembled, can activate caspase-1 and subsequently process and secrete interleukin (IL)-1β [22]. The CT genotype of *NLRP3* rs34298354, Ex4-849C/T, is associated with an increased risk of silicosis (OR = 2.4). Furthermore, carrying the TT genotype of *NLRP3* rs1539019 G/T is associated with an increased risk of CWP (OR = 1.39), particularly among smokers (OR = 1.67) [23, 24]. However, it is currently unclear whether NLRP3 and NLRC4 play a role in the development and progression of CWP.

In this study, we selected 14 SNP sites of *ATM*, *ERCC1*, *XRCC1*, *NLRP1*, *NLRP3*, and *NLRC4* genes based on the minimum allele frequency (typically >0.05) to investigate their association with the development and progression of CWP in Han Chinese male subjects. Epidemiological data of the study subjects were also analysed to identify factors that may affect CWP development. Furthermore, the protein expression levels of mutated genes in plasma were observed to comprehensively evaluate the potential of proteins to act as early warning and diagnostic biomarkers for CWP.

## Materials and methods

### SNP selection and genotyping of study subjects

According to the method of the target value in the US FDA’s review of medical devices [25, 26] and combined with the actual receipt of samples, of the 554 randomly selected Han Chinese subjects, 131 healthy individuals (the person is not coal workers and is no coal dust exposure.) were included in the healthy group, 140 coal miners were included in the coal dust exposure group, 141 patients with stage I CWP were selected as the stage I CWP group, and 142 patients with stage II CWP were selected as the stage II CWP group. All study subjects were male. Two milliliters of EDTA anticoagulated blood were collected from each participant between January 2020 and December 2022. The Ethics Committee of Guiyang Public Health Clinical Center approved the study (approval no. 201920). All patients provided written informed consent to participate in this study. Epidemiological data, including age, work duration, and smoking, drinking, and medical history, were collected using questionnaires. Patients with other lung diseases, such as tuberculosis and lung cancer, as well as autoimmune diseases, were excluded from this study.

Fourteen SNP sites, including *ATM* (rs201159454, rs2234997, and rs30928443), *ERCC1* (rs3212929 and rs3212986), *XRCC1* (rs25489 and rs915927), *NLRP1* (rs2301582 and rs12150220), *NLRP3* (rs3806268 and rs10802501), and *NLRP4* (rs6757121, rs455060, and rs408813), were selected based on previous literature and a minimum allele frequency >0.05. The genotype distribution of these SNP sites all complied with the Hardy–Weinberg equilibrium (P>0.05). Two millilitres of EDTA anticoagulated blood were collected from each study subject, and genomic DNA was extracted from the blood using a blood genomic DNA extraction kit. The genotyping of all study subjects was performed using MassARRAY SNP genotyping technology.

### Measurement of ERCC1 in plasma

Whole blood samples (2 mL) of an additional 30 study subjects were collected randomly in EDTA anticoagulant tubes and centrifuged for 5 min at 3,500 rpm to obtain plasma supernatant. The enzyme-linked immunosorbent assay kit was selected based on genotyping information; mutated gene expression levels in the plasma were calculated according to the manufacturer’s instructions.

### ERCC1 pathways analysis based on multi-omics

Five groups including healthy group (n = 32), currently receiving dust group (n = 33), previously receiving dust group (n = 32), CWP stage I (n = 34), CWP stage II (n = 26) were constructed. Whole blood samples (5 ml) were collected in heparin anticoagulant tube and centrifuged for 5 min at 3,500 rpm to obtain the plasma supernatant.

### Metabolism analysis

Plasma sample containing 10 μL 0.3 mg/mL of 2-chloro-l-phenylalanine as internal standard and150 μL methanol and acetonitrile (2V/1V) was mixed, vortexed, ultrasonicated and precipitated at -20°C for 10 min, and centrifuged at 14000 rpm at 4°C for 10 min. The 150 μL supernatants were used to be analysed by UPLC–Q-TOF/MS (Waters Vion IMS Qtof)).

### Proteomic analysis

The samples in each group were selected at random with 10 cases and mixed, and each group was represented by three biological replicates. The total serum samples (n = 15) were analysed.

EasyDeeP Sample Preparation kit (OSFP0002) was used to analysis plasma according to protocols provided. Mainly,100 μL plasma and 1mg beads mixed and incubated at 37°C for 1 h, and beads were washed to obtain the precipitate with wash solution buffer. The precipitate was digested, collected and desalted to obtain the peptides. The peptides were re-dissolved in solvent A (A: 0.1% formic acid in water) and analyzed by by UPLC–Q-TOF/MS (Orbitrap Fusion™ Tribrid™ coupled to an EASY-nanoLC 1200 system (Thermo Fisher Scientific, MA, USA)). The mass spectrometer was run under data independent acquisition (DIA) mode with hybrid data strategy.

### Bioinformation analysis

The UPLC–Q-TOF/MS raw data were analyzed by progenesis QI (Waters Corporation, Milford, USA) software. Including m/z, peak RT and peak intensities, and RT–m/z pairs were used as the identifier for each ion. The different metabolites standard was variable important in projection (VIP)>1. KEGG enrichment was analyzed with different metabolites. The pathways overview diagram was drawed using MetaboAnalyst 4.0.

Data of DIA were processed and analyzed by Spectronaut 15.0 (Biognosys AG, Switzerland) with default settings. Spectronaut was set up to search the database of uniprot-homo sapiens (version201907, 20428 entries). The different expressed proteins were selected if their p adj value <0.05 and absolute fold change >1.5. Blast2GO version 5 was used for functional annotation and GOATOOLS was used to perform GO enrichment analysis. Pathway analysis was processed by KOBAS (http://kobas.cbi.pku.edu.cn/). Protein-protein interaction network was constructed by using STRING v10 (www.string-db.org).

### Statistical analysis

Statistical analysis was performed using SPSS 23.0 software. Quantitative data that followed a normal distribution are expressed as $\bar{x}$±S, and one-way ANOVA was used for comparison. Count data were compared using the χ2 test of the R×C contingency table. Multinomial logistic regression analysis was used to adjust the OR and 95% confidence interval (CI). The optimal cutoff value of the protein marker was determined by receiver operating characteristic (ROC) curve analysis, and the accuracy of the biomarker was evaluated based on the corresponding area under the curve (i.e., >0.700, 0.700–0.900 and >0.900), sensitivity, specificity, and Youden index. P<0.05 was considered significant in all analyses.

## Results

### Basic status of subjects

A total of 554 subjects were included in this study. The basic clinical information of the study subjects is presented in Table 1.

**Table 1:** Analysis of basic clinical data of the study population ($\bar{x}$± s).

Variable	Group			
	Healthy group (n = 131)	Coal dust exposure group (n = 140)	Stage I CWP group (n = 141)	Stage II CWP group (n = 142)
Age (y)	31.01±8.45	48.96±5.28	50.97±9.28	54.69±9.57
Work duration (y) (year)	0	19.55±8.11	19.30±7.31	17.72±7.44
Smoking history:				
never smoked	54 (41.2%)	49 (35.0%)	69 (48.9%)	55 (38.7%)
occasional smoker	19 (14.5%)	13 (9.3%)	10 (7.1%)	20 (14.1%)
regular smoker	58 (44.3%)	78 (55.7%)	62 (44.0%)	67 (47.8%)
Drinking history				
never drank	71 (54.2%)	69 (49.3%)	98 (69.5%)	76 (53.5%)
occasional drinker	59 (45.0%)	62 (44.3%)	37 (26.2%)	54 (38.0%)
regular drinker	1 (0.8%)	9 (6.4%)	6 (4.3%)	12 (8.5%)

Note: numbers in parentheses indicate the composition percentage (%).

### Change in genotypes with CWP progression

The SNP genotypes of *ATM* (rs201159454, rs2234997, and rs30928443), *ERCC1* (rs3212929 and rs3212986), and *XRCC1* (rs25489 and rs915927) in the study subjects are listed in Table 2. As CWP developed, we observed a decrease in the CC genotype and an increase in the CA genotype of *ERCC1* rs3212986 (P<0.05). However, the genotypes of *ATM* (rs201159454, rs2234997, and rs30928443), *ERCC1* rs3212929, and *XRCC1* (rs25489 and rs915927) showed no change with CWP progression (P > 0.05). The SNP genotypes of *NLRP1* (rs2301582 and rs12150220), *NLRP3* (rs3806268 and rs10802501), and *NLRP4* (rs6757121, rs455060, and rs408813) in the study subjects are listed in Table 3. None of the genotypes exhibited any change with CWP progression (P > 0.05).

**Table 2:** Change in the genotypes of *ATM*, *ERCC1*, and *XRCC1*.

Gene	SNP	Healthy group		Coal dust exposure group		Stage I CWP group		Stage II CWP group		P
		n	%	n	%	n	%	n	%	
ATM	rs201159454									*
	TT	131	100	140	100	141	100	142	100	
	rs2234997									*
	TT	131	100	140	100	141	100	142	100	
	rs3092844									*
	CC	131	100	140	100	141	100	142	100	
ERCC1	rs3212929									*
	CC	131	100	140	100	141	100	142	100	
	rs3212986									
	CC	55	42.0	60	42.9	49	34.8	39	27.5	0.031
	AA	22	16.8	20	14.3	16	11.3	30	21.1	
	CA	54	41.2	60	42.9	76	53.9	73	51.4	
XRCC1	rs25489									
	TT	2	1.5	1	0.7	2	1.4	2	1.4	0.993
	CC	105	80.2	116	82.9	116	82.3	117	82.4	
	TC	24	18.3	117	16.4	23	16.3	23	16.2	
	rs915927									
	CC	1	0.8	0	0	3	2.1	3	2.1	0.121
	TT	106	81.5	109	77.9	109	77.3	97	68.3	
	CT	23	17.7	31	22.1	29	20.6	42	29.6	

Note: * indicates that the statistical measure cannot be calculated.

**Table 3:** Changes in the genotypes of *NLRP1*, *NLRP3*, and *NLRP4*.

Gene	SNP	Healthy group		Coal dust exposure group		Stage I CWP group		Stage II CWP group		P
		n	%	n	%	n	%	n	%	
NLRP1	rs2301582									0.690
	TT	0	0	1	0.7	0	0	0	0	
	CC	125	95.4	133	95.0	132	93.6	136	95.8	
	TC	5	4.6	6	4.3	9	6.4	6	4.2	
	rs12150220									0.126
	AA	125	95.4	136	97.1	129	91.5	137	96.5	
	TT	0	0	1	0.7	0	0	0	0	
	AT	6	4.6	3	2.1	12	8.5	5	3.5	
NLRP3	rs3806268									0.589
	GG	31	23.7	33	23.6	45	31.9	40	28.2	
	AA	40	30.5	37	26.4	38	27.0	35	24.6	
	GA	60	45.8	70	50.0	58	41.1	67	47.2	
	rs10802501									
	AA	0	0	0	0	1	0.7	0	0	0.270
	TT	123	93.9	131	93.6	134	95.0	127	89.4	
	AT	8	6.1	9	6.4	6	4.3	15	10.6	
NLRP4	rs6757121									
	TT	3	2.3	2	1.4	3	2.1	1	0.7	0.454
	CC	105	80.2	111	79.3	122	86.5	122	85.9	
	TC	23	17.6	27	19.3	16	11.3	19	13.4	
	rs455060									
	GG	52	39.7	50	35.7	43	30.5	50	35.2	0.758
	AA	26	19.8	25	17.9	27	19.1	25	17.6	
	GA	53	40.5	65	46.4	71	50.4	67	47.2	
	rs408813									*
	GG	131	100	140	100	141	100	142	100	

Note: * indicates that the statistical measure cannot be calculated.

### Effects of ERCC1 rs3212986 on the risk of CWP

Multiple-factor multinomial logistic regression analysis was conducted on the groups based on the development and progression of CWP as the dependent variable, and age, work duration, smoking, alcohol drinking, and the *ERCC1* rs3212986 genotype as independent variables (see Table 4). The goodness-of-fit was P < 0.05. Compared to the CC genotype in the rs3212986 locus, significant differences (P<0.05) were observed in the AA (OR = 3.016, 95% CI = 1.409–6.456, P<0.05) and CA (OR = 2.130, 95% CI = 1.202–3.776, P<0.05) genotypes in patients with stage II CWP. Compared to workers in the coal dust exposure group, significant differences (P<0.001) in age were observed in patients with stage I CWP (OR = 1.047, 95% CI = 1.008–1.088, P<0.05) and stage II CWP (OR = 1.1267, 95% CI = 1.084–1.171, P<0.05). Compared to workers in the coal dust exposure group, significant differences (P<0.001) in work duration were observed in patients with stage II CWP (OR = 0.924, 95% CI = 0.890–0.959, P<0.05).

**Table 4:** Regression model analysis of the influence of *ERCC1* rs3212986 on CWP.

Variable	Stage I CWP group			Stage II CWP group		
	OR	95%CI	p	OR	95%CI	p
Age	1.047	1.008–1.088	P<0.05	1.1267	1.084–1.171	P<0.001
Work duration (year)	0.983	0.950–1.017	0.316	0.924	0.890–0.959	P<0.001
Never smoked*						
Occasional smoker	0.828	0.322–2.133	0.696	2.312	0.943–5.666	0.067
Regular smoker	0.800	0.465–1.378	0.421	1.054	0.580–1.917	0.863
Never drank*						
Occasional drinker	0.466	0.269–0.808	0.007	0.720	0.404–1.282	0.264
Regular drinker	0.475	0.154–1.462	0.194	0.940	0.326–2.716	0.910
CC*						
AA	1.164	0.533–2.542	0.704	3.016	1.409–6.456	P<0.05
CA	1.593	0.947–2.681	0.079	2.130	1.202–3.776	P<0.05

Note: * indicates the control.

To explore the interaction effect of these factors, further interactive analysis was conducted using binary logistic regression analysis, with dust-exposed workers with or without CWP as the dependent variable and rs3212986, smoking, and drinking, as well as their interactions, as independent variables. The interaction of smoking and drinking (OR = 1.787, 95% CI = 1.251–2.552, P < 0.05) increased the risk of CWP with a synergistic effect when rs3212986 was not included. When rs3212986 was included, smoking, drinking, and *ERCC1* rs3212986 all increased the risk of CWP independently (OR>1). However, the interaction of rs3212986 and smoking (OR = 0.527, 95% CI = 0.324–0.858, P < 0.05) and the interaction of rs3212986 and drinking (OR = 0.769, 95% CI = 0.559–1.058, P < 0.05) reduced the risk of CWP with an antagonistic effect (see Table 5).

**Table 5:** Effect of the interaction of drinking, smoking and *ERCC1* rs3212986 on workers exposed to coal dust.

Variable	B	β	Wald	P	OR (95% CI)
Smoking	−1.814	0.614	8.745	0.003	0.163 (0.049–0.542)
Drinking	−1.607	0.595	7.302	0.007	0.200 (0.062–0.643)
Smoking × Alcohol Drinking	0.580	0.182	10.195	0.001	1.787 (1.251–2.552)
rs3212986	1.768	0.811	4.747	0.029	5.858 (1.194–28.730)
Smoking	2.274	0.831	7.485	0.006	9.713 (1.906–49.513)
rs3212986 × Smoking	−0.640	0.248	6.650	0.010	0.527 (0.324–0.858)
Drinking	1.146	0.540	4.496	0.034	3.144 (1.091–9.066)
rs3212986	0.450	0.509	0.783	0.376	1.569 (0.578–4.255)
rs3212986 × Drinking	−0.263	0.163	2.605	0.107	0.769 (0.559–1.058)

### Plasma concentrations and ROC curve analysis of the ERCC1 protein in CWP

**Fig 1:**
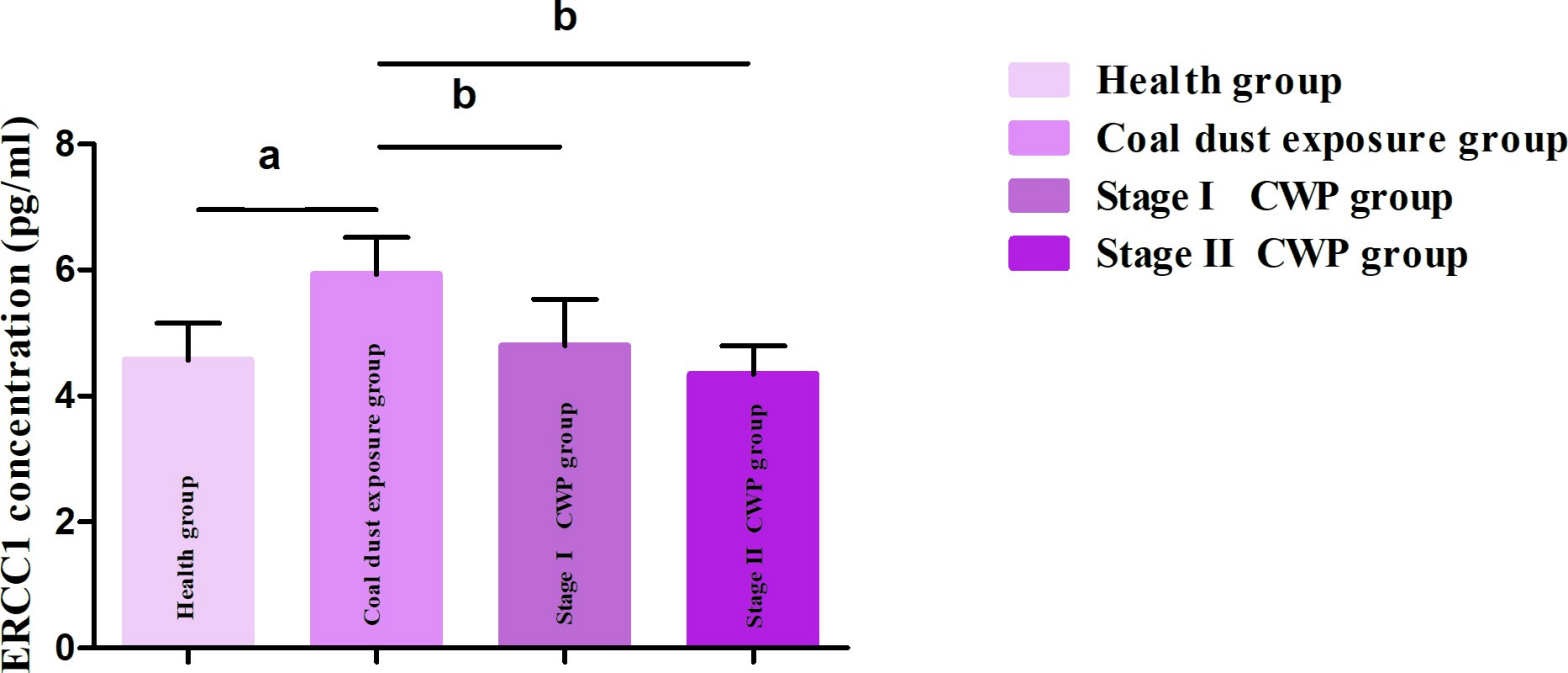
Change in the ERCC1 secretion level in plasma(pg/ml). Note: a indicates a significant difference compared to the healthy group (P<0.05); b indicates a significant difference compared to the coal dust exposure group.

**Fig 2:**
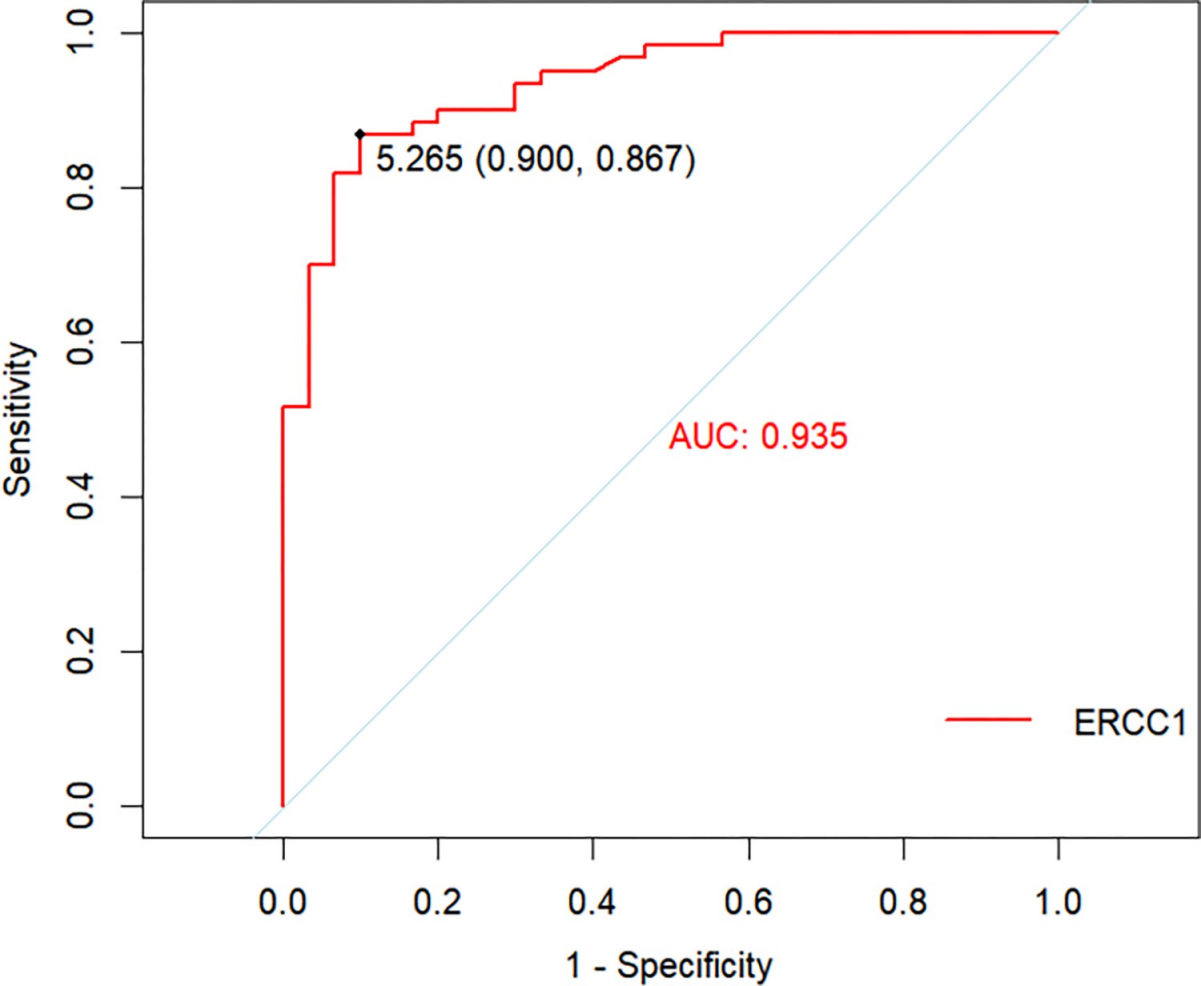
ROC curve analysis for ERCC1 with CWP progression.

Plasma ERCC1 protein levels differed among the five groups (P < 0.05). Compared to the healthy group, the coal dust exposure group showed elevated ERCC1 levels (P < 0.05). Compared to the coal dust exposure group, stage I and stage II CWP groups showed decreased ERCC1 levels (P < 0.05) (Fig 1). ROC curve analysis was conducted using the ERCC1 level in the plasma of study subjects as the test variable, and the results showed that the area under the curve was 0.935. The sensitivity was 90.0% and the specificity was 86.7%, indicating a higher value for early warning and diagnosis. Thus, ERCC1 can be used as an auxiliary indicator for the early warning and diagnosis of CWP, and the cut-off value for early warning and diagnosis of the disease is 5.265 pg/ml (Fig 2).

### Pathways of ERCC1

**Fig 3:**
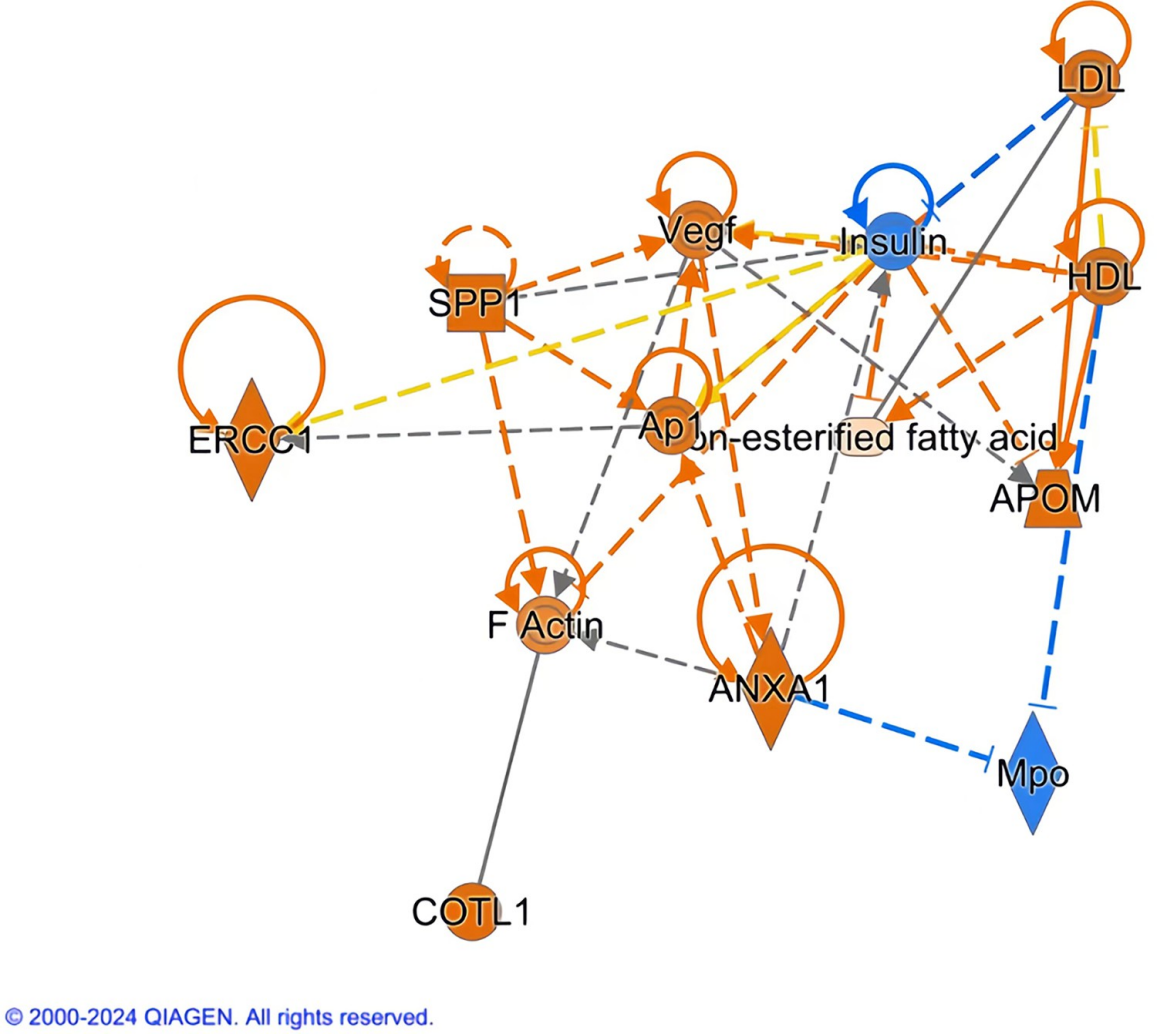
Pathways analysis of ERCC1 based on CWP metabolome and proteome. The arrows in the figure indicate that the proteins interact, and there is no upstream-downstream relationship. Dashed lines mean indirect relationship and solid lines mean direct relationship.

Briefly, ERCC1, osteopontin (SPP1), annexin A1(ANXA1), low-density lipoprotein (LDL), high-density lipoprotein (HDL), apolipoprotein M(APOM), vascular endothelial growth factor (Vegf), coactosin-like protein (COTL1) (that is binding proteins of actin.), activator protein-1(Ap1), and polymeric filamentous actin (F-actin) had strongly activated and non-esterified fatty acid was less active. But insulin and Mpo (Myeloperoxidase) were inhibited. ERCC1 had indirect interaction with insulin and Ap-1 and the change between ERCC1 and insulin was opposite. Inhibited insulin as an import core had an indirect interaction with LDL, HDL, APOM, non-esterified fatty acid, ANXA1 and Vegf. Between insulin and LDL was an inhibitive interaction and between Insulin and Vegf had an interaction of inconsistment with state of downstream molecule. The rest interactions from insulin were activated. Active Ap1 as a core had an indirect and active interaction with SPP1, ANXA1 and Vegf indirectly and it had two types of interaction with insulin including active effect and inconsisitment effect with state of downstream molecule. The remaining indirect interactions of Vegf were with SPP1, F-actin, ANXA, APOM and HDL. There was a direct interaction between F-Actin and COTL1. APOM had direct and active interactions with LDL and HDL. LDL had a direct interaction with non-esterified fatty acid but it had an indirect and inconsisitment with state of downstream molecule interaction with HDL. SPP1 had indirect interaction with F-actin and ANXA1 had indirect interaction F-actin. Mpo had Inhibited interactions with ANXA1 and HDL (Fig 3).

## Discussion

Dust entering the body through the respiratory tract triggers oxidative stress reactions, which first damage mitochondrial DNA in cells [27]. The body then activates the DNA damage repair system. Meanwhile, mitochondrial DNA mediates the NLRP3 pathway, which in turn activates inflammasomes [28]. Based on DNA damage repair pathways, previous research identified a significant mutation in *ATM* rs189037, which is a key gene involved in double-stranded DNA repair [15], and identified ATM as an early warning and diagnostic biomarker for CWP [29]. In the present study, based on the NER pathway of DNA damage repair, we identified for the first time that the *ERCC1* rs3212986 CA and AA genotypes are risk factors for the development and progression of CWP (OR>1). Previous studies have found that *ERCC1* rs3212986 is a genetic susceptibility factor for lung cancer and is associated with a reduced overall survival rate in patients with late-stage non-small cell lung cancer and decreased activity of the chemotherapy drug cisplatin [30–35]. In addition, rs3212986 alters the DNA damage repair capacity of the body by regulating ERCC1 expression. rs3212986 is a risk factor for smoking-related lung cancer and may also be an indicative biomarker [36]. In addition to smoking, alcohol consumption in men living in highly polluted areas is a risk factor for lung cancer [37]. The results of the present study demonstrated that, after exposure to coal dust, *ERCC1* rs3212986 is prone to mutation, which might reduce its expression and the DNA damage repair capacity, ultimately increasing susceptibility to CWP. ROC curve analysis revealed a decrease in plasma ERCC1 protein levels with disease progression, indicating its potential value as an early warning and diagnostic biomarker. This may be associated with the positive DNA damage repair function of ERCC1 in the development and progression of CWP. Subjects in the coal dust exposure group exhibited the highest levels of ERCC1, indicating an increased DNA repair capacity at this stage after dust exposure, which may be advantageous in inhibiting the occurrence and development of CWP. However, as the disease progressed, plasma ERCC1 levels significantly decreased in patients with stage I and stage II CWP, to a level comparable to that of the control group, suggesting that the body lost its DNA damage repair capacity, which led to the final stage of the disease. Therefore, effective biomarkers should be used for real-time monitoring of the health status of workers exposed to dust in the early stages, so that early detection and control measures can be taken to alleviate disease progression and change the outcome of the disease. According to this study, when the plasma concentration of ERCC1 in individuals exposed to dust exceeds the cut-off value of 5.265 pg/mL, they are at risk of developing CWP and intervention should be performed. When the cut-off value is reached, individuals exposed to dust should promptly discontinue contact with the dust and receive regular follow-up observations to determine subsequent disease detection and control strategies.

In the logistic regression model, smoking and drinking interaction increased the risk of CWP in individuals exposed to dust when *ERCC1* rs3212986 was not included; however, smoking and drinking alone had an antagonistic effect. When rs3212986 was included, rs3212986, smoking, and drinking all increased the risk of disease individually. However, when the interactions with smoking or drinking were considered, the OR value of rs3212986 decreased significantly, indicating an antagonistic effect. The possible reasons for this phenomenon may be related to whether different types of dust cause lung cancer; unfortunately, the mechanism of dust-induced carcinogenesis is currently not well understood. It is generally believed that asbestos is a carcinogen that causes lung cancer, exhibiting a synergistic interaction with tobacco in lung cancer [38]. Silica is usually considered a carcinogen and has a synergistic interaction with tobacco [39], although this is not always the case. Compared with subjects exposed to dust but not exhibiting silicosis, patients with silicosis have a higher risk of developing lung cancer. However, the carcinogenic effect of silica itself is controversial for exposed populations without silicosis [40]. Smoking increases the incidence of lung cancer compared to that in subjects without CWP, but CWP status increases the incidence of cancer in subjects who do not smoke. In addition, CWP is associated with a decrease in the incidence of non-lung cancer [41]. The incidence of small cell carcinoma is three times higher in the non-CWP population than in the CWP population and is associated with smoking in the non-CWP population [42]. These findings indicate that coal dust exposure may not be related to the risk of lung cancer and may show no synergistic interaction with smoking. Therefore, the role of rs3212986 in CWP may be different from its role in lung cancer, and the environmental risk factors of smoking and drinking may also play different roles in CWP and in lung cancer. Age and work duration may indirectly affect the level of DNA damage repair in the body following exposure to coal dust. With increasing age, the DNA damage repair capacity of the body gradually decreases [43], and the risk of CWP correspondingly gradually increases, making age a risk factor for the disease. The longer the duration of work, the longer the body is exposed to dust, and the greater the DNA damage caused by dust, which reduces the DNA damage repair capacity of the body and leads to CWP. Conversely, the shorter the duration of work, the shorter the exposure time to dust, and the lesser the DNA damage caused by dust. The body has a relatively high capacity for DNA damage repair, and the risk of developing the disease decreases. Therefore, the duration of work is a protective factor for CWP.

Based on multi-omics analysis of serum proteome and metabolome of IPA database, we found that ERRC1 played a much more important role in lipid metabolism and actin dynamics. ERCC1 pathways are mainly composed of non-esterified fatty acid, proteins involved in metabolizing lipids including LDL, HDL, APOM and other proteins that affect actin dynamics. ERCC1 may affect lipid metabolism by the pathways of Ap1 and insulin. Lipid peroxidation (LPO) is a product of oxidative damage and can react with DNA. High concentration LPO inhibits NER and BER and down-regulates DNA repair and up-regulates DNA damage. Mouse embryonic fibroblasts and mice with ERCC1-XPF deficient were hypersensitive to LPO and levels of DNA damage were increased [44–47]. Therefore, ERCC1 level decreased and LPO concentration increased with CWP development. LPO-induced DNA lesion level was higher and DNA repair level was lower. The above results had already confirmed ERCC1 rs3212986 was a risk factor and ERCC1 alteration had diagnosis value. The role of ERCC1 in regulating lipids metabolism further provided further evidence that DNA repair inhibits CWP development. Ap1 regulates various biology responses and is associated with serve diseases including fibrosis, organ injury, and various inflammatory disorders. The targets of Ap1 singaling comprise DNA, histone and protein methyltransferases [48]. The relationship between ERCC1and Ap1 suggests Ap1 may take part in CWP development and play multiple roles such as fibrosis, inflammatory disorders and so on. ERCC1 has a negative impact on pancreatic beta-cell and causes the increasement of insulin sensitivity [49]. Lipid intermediates made with free fatty acids and lipid droplet leads to down -regulates insulin signaling pathways and glucose metabolic disorders [50]. Hence, ERCC1 may affect the insulin metabolism in patients with CWP mediated via its impact on lipids metabolism. SPP1 and ANXA1 is associated with pneumoconiosis and they may be the disease biomarkers [51, 52]. Mpo is a peroxidase which takes part in oxidative stress response [53] and thus it may involve in the CWP development. The actin cytoskeleton reorganization is a response to TGF-β1 singaling early. Rho-like GTPases (RhoB) and α-SMA in fibroblasts are active because long-term actin cytoskeleton reorganization activates the transforming growth factor β-SMAD (TGFβ-SMAD) pathway so that actin dynamics affects the pathological process such as wound repair, inflammation and so on [54]. The correction both actin and TGF-β1 suggests that actin dynamics change may be an indicative marker of fibrosis. On the other hand, when oxidative stress and inflammatory injures endothelial cells, the endothelial-mesenchymal transition (E(nd)MT) will happen. E(nd)MT leads to fibrosis and then Vegf is increased [55]. Similarly, the dust stimulation induces the same injury of endothelial cells, and causes fibrosis and high level Vegf. The interaction among Vegf, insulin and Ap1 confirms that ERCC1 may alleviate abnormal changes in endothelial cells by affecting the pathways of insulin and Ap1. Above all, ERCC1 may play a critical role in inhibiting CWP fibrosis though changing lipids metabolism and actin dynamics. ERCC1 has been studied mainly in the field of cancer, focusing on the relationship between its polymorphisms and the development of cancers, such as lung cancer. The reason is relative with its function of DNA repair. However, its association with human pulmonary fibrosis disease has not been studied so far. In this paper, we propose for the first time that ERCC1 polymorphisms are risk factors for CWP and find it has much better sensitivity, specificity and provide the cut-off value for early detection and intervention.

Given the relatively small sample size of this study, it is necessary to increase the sample size in future studies to verify the changing patterns of rs3212986 and ERCC1 pathways based on proteome and metabolome. Meanwhile, there was a difference between SNP analysis and multi-omics analysis in the divided groups. Because the sample size of currently receiving dust group and previously receiving dust group alone was not enough to be analysed with SNP, the two groups were combined the coal dust exposure group. Therefore, it should collect much more samples of currently receiving dust and previously receiving dust group for better SNP analysis of CWP, respectively. Additionally, further studies are required to investigate whether other SNP sites of the six genes affect the development of CWP. In this study, we did not find any interaction between the six genes involved in DNA damage repair and inflammatory response in CWP. Therefore, other important genes that affect DNA damage repair and the inflammatory response should be studied. Furthermore, environmental factors such as smoking and drinking have a certain impact on the disease outcome in combination with susceptible genes and different types of dust, and many confounding factors exist, leading to heterogeneity in research findings. However, the current lack of research in this area highlights the need to further clarify this issue. Finally, future research is required to investigate the key molecules involved in the pathway to ascertain mechanisms of ERCC1 on CWP development and progression and its interaction with environmental factors.

### Conclusions

In this study, we investigated the polymorphisms of SNP sites in genes closely related to DNA damage repair and the inflammatory response involved in the development of CWP. The results showed that *ERCC1* rs3212986 is a risk factor for CWP. Moreover, the plasma level of ERCC1 decreased with CWP development, indicating its potential as a biomarker for early warning and diagnosis. Age and work duration indirectly affected the DNA damage repair capacity of the body. Finally, we observed an antagonistic interaction between rs3212986 and environmental factors including smoking and drinking. This study provides an effective biomarker for the real-time monitoring of the health status of workers exposed to dust and will help develop a more precise intervention for CWP progression. When ERCC1 concentration reaches cut-off value, workers should leave the workplace and change other jobs and will failure to develop and diagnose pneumoconiosis. ERCC1 could be used for regular screening and at the sametime reduction of working hours, no smoking, improvement in air ventilation, better personal protection equipment (PPE) for the workers are workable and excellent measures. For healthy coal workers, the cohort studies should be carried on using ERCC1, working hours and jobs to build the connection of them and to find a cut-off value of working hours. Early detection and interventions should be carried in factory using the threshold to decrease morbidity of CWP and the burden of disease. The alteration and mechanism of ERCC1 in CWP development should be studied in the animal models and cell models exposed to different coal dust dosages to build the link between the two to provide reference for clinical. The study provides a useful judgement criteria for early detection and invention. Applying it wisely could raise the attention of employers to the hazards of occupational diseases, enhance workers’ awareness of self-protection, and promote the harmonious development of society as a whole. The application of ERCC1 will facilitate the strengthening administrative supervision and management, the promotion of industry self-regulation, and the improvement of the diagnostic and treatment level of occupational disease examination and diagnosis institutions.

## Supporting information

S1 Data (XLSX)

## References

[1] Li ZG, Li BC, Li ZW, Hu HY, Ma X, Cao H, et al. The Potential Diagnostic Biomarkers for the IgG Subclass in Coal Workers’ Pneumoconiosis. J Immunol Res. 2023 Mar 14;2023:9233386. doi: 10.1155/2023/9233386 36959921 PMC10030223

[2] Li J, Yin P, Wang H, Wang L, You J, Liu J, et al. The burden of pneumoconiosis in China: an analysis from the Global Burden of Disease Study 2019. BMC Public Health. 2022 Jun 3;22(1):1114. doi: 10.1186/s12889-022-13541-x 35659279 PMC9166455

[3] United Nations (2015). 2015b. Transforming our world: The 2030 Agenda for Sustainable 488 Development. Resolution adopted by the General Assembly on 25 September 2015. A/RES/70/1. 489 UN General Assembly, Seventieth Session. Agenda items 15 and 116. New York: United Nations.

[4] General Office of the State Council, China (2016). National Plan for Prevention and Control of 491 Occupational Diseases (2016–2020), 26 December 2016 (Guo Ban Fa [2016] No:100).

[5] Li Y, Cheng Z, Fan H, Hao C, Yao W. Epigenetic Changes and Functions in Pneumoconiosis. Oxid Med Cell Longev. 2022 Jan 20;2022:2523066. doi: 10.1155/2022/2523066 35096264 PMC8794660

[6] Hou Z, Zhang X, Gao Y, Geng J, Jiang Y, Dai H, et al. Serum Osteopontin, KL-6, and Syndecan-4 as Potential Biomarkers in the Diagnosis of Coal Workers’ Pneumoconiosis: A Case-Control Study. Pharmgenomics Pers Med. 2023 Jun 1;16:537–549. doi: 10.2147/PGPM.S409644 37284491 PMC10241210

[7] Jomova K, Raptova R, Alomar SY, Alwasel SH, Nepovimova E, Kuca K, et al. Reactive oxygen species, toxicity, oxidative stress, and antioxidants: chronic diseases and aging. Arch Toxicol. 2023 Oct;97(10):2499–2574. doi: 10.1007/s00204-023-03562-9 37597078 PMC10475008

[8] Seyedsadjadi N, Grant R. The Potential Benefit of Monitoring Oxidative Stress and Inflammation in the Prevention of Non-Communicable Diseases (NCDs). Antioxidants (Basel). 2020 Dec 27;10(1):15. doi: 10.3390/antiox10010015 33375428 PMC7824370

[9] Makena P, Kikalova T, Prasad GL, Baxter SA. Oxidative Stress and Lung Fibrosis: Towards an Adverse Outcome Pathway. Int J Mol Sci. 2023 Aug 6;24(15):12490. doi: 10.3390/ijms241512490 37569865 PMC10419527

[10] Barber C, Fishwick D (2016) Pneumoconiosis. Medicine. 2016 Mar 1;44(6): 355–358.

[11] Chair SY, Chan JYW, Law BMH, Waye MMY, Chien WT. Genetic susceptibility in pneumoconiosis in China: a systematic review. Int Arch Occup Environ Health. 2023 Jan;96(1):45–56. doi: 10.1007/s00420-022-01893-1 35906431

[12] Anlar HG, Bacanli M, Kurt ÖK, Eraydin C. DNA damage assessment with buccal micronucleus cytome assay in Turkish coal miners. Arh Hig Rada Toksikol. 2019 Dec 1;70(4):283–289. doi: 10.2478/aiht-2019-70-3332 32623860

[13] León-Mejía G, Luna-Rodríguez I, Trindade C, Oliveros-Ortíz L, Anaya-Romero M, Luna-Carrascal J, et al. Cytotoxic and genotoxic effects in mechanics occupationally exposed to diesel engine exhaust. Ecotoxicol Environ Saf. 2019 Apr 30;171:264–273. doi: 10.1016/j.ecoenv.2018.12.067 30612014

[14] Da Silva Pinto EA, Garcia EM, de Almeida KA, Fernandes CFL, Tavella RA, Soares MCF, et al. Genotoxicity in adult residents in mineral coal region-a cross-sectional study. Environ Sci Pollut Res Int. 2017 Jul;24(20):16806–16814. doi: 10.1007/s11356-017-9312-y 28567685

[15] Deng H, Zhang T, Wu ML, Yang GG, Chen Y, Liang YD. [Expression and functional SNP loci screen of ATM from coal worker’s pneumoconiosis]. Zhonghua Lao Dong Wei Sheng Zhi Ye Bing Za Zhi. 2022 Feb 20;40(2):103–108. Chinese. doi: 10.3760/cma.j.cn121094-20201019-00590 35255575

[16] Kell L, Simon AK, Alsaleh G, Cox LS. The central role of DNA damage in immunosenescence. Front Aging. 2023 Jul 3;4:1202152. doi: 10.3389/fragi.2023.1202152 37465119 PMC10351018

[17] Weilbeer C, Jay D, Donnelly JC, Gentile F, Karimi-Busheri F, Yang X, et al. Modulation of ERCC1-XPF Heterodimerization Inhibition via Structural Modification of Small Molecule Inhibitor Side-Chains. Front Oncol. 2022 Mar 17;12:819172. doi: 10.3389/fonc.2022.819172 35372043 PMC8968952

[18] London RE. XRCC1—Strategies for coordinating and assembling a versatile DNA damage response. DNA Repair (Amst). 2020 Sep;93:102917. doi: 10.1016/j.dnarep.2020.102917 33087283 PMC7587305

[19] Clementi E, Inglin L, Beebe E, Gsell C, Garajova Z, Markkanen E. Persistent DNA damage triggers activation of the integrated stress response to promote cell survival under nutrient restriction. BMC Biol. 2020 Mar 30;18(1):36. doi: 10.1186/s12915-020-00771-x 32228693 PMC7106853

[20] Rastrick J, Birrell M. The role of the inflammasome in fibrotic respiratory diseases. Minerva Med. 2014 Feb;105(1):9–23. 24572449

[21] Song MY, Wang JX, Sun YL, Han ZF, Zhou YT, Liu Y, et al. Tetrandrine alleviates silicosis by inhibiting canonical and non-canonical NLRP3 inflammasome activation in lung macrophages. Acta Pharmacol Sin. 2022 May;43(5):1274–1284. doi: 10.1038/s41401-021-00693-6 34417574 PMC9061833

[22] Lam M, Mansell A, Tate MD. Another One Fights the Dust: Targeting the NLRP3 Inflammasome for the Treatment of Silicosis. Am J Respir Cell Mol Biol. 2022 Jun;66(6):601–611. doi: 10.1165/rcmb.2021-0545TR 35290170

[23] Weng S, Wang L, Rong Y, Liu Y, Wang X, Guan H, et al. Effects of the Interactions between Dust Exposure and Genetic Polymorphisms in Nalp3, Caspase-1, and IL-1β on the Risk of Silicosis: A Case-Control Study. PLoS One. 2015 Oct 23;10(10):e0140952. 26496436 10.1371/journal.pone.0140952 PMC4619690

[24] Ji X, Hou Z, Wang T, Jin K, Fan J, Luo C, et al. Polymorphisms in inflammasome genes and risk of coal workers’ pneumoconiosis in a Chinese population. PLoS One. 2012;7(10):e47949. doi: 10.1371/journal.pone.0047949 23110140 PMC3478280

[25] Xu Y, Li H, Hedmer M, Hossain MB, Tinnerberg H, Broberg K, et al. Occupational exposure to particles and mitochondrial DNA—relevance for blood pressure. Environ Health. 2017 Mar 9;16(1):22. doi: 10.1186/s12940-017-0234-4 28274239 PMC5343309

[26] Food and Drug Administration Center for Devices and Radiological Health. Clinical study designs for Catheter Ablation Devices for treatment of atrial flutter (Guidance for industry andFDA staff) [EB/OL]. Available from https://www.fda.gov/media/71116/download

[27] Park SR, Kong SY, Nam BH, Choi IJ, Kim CG, Lee JY, et al. CYP2A6 and ERCC1 polymorphisms correlate with efficacy of S-1 plus cisplatin in metastatic gastric cancer patients. Br J Cancer. 2011 Mar 29;104(7):1126–34. doi: 10.1038/bjc.2011.24 21364592 PMC3068488

[28] Fang C, Wei X, Wei Y. Mitochondrial DNA in the regulation of innate immune responses. Protein Cell. 2016 Jan;7(1):11–6. doi: 10.1007/s13238-015-0222-9 26498951 PMC4707157

[29] Tao Z, MaLi W, Hao D et al. (2023) Application of ATM protein in the preparation of pneumoconiosis diagnosis products. South African patents:2023/04255

[30] Pintarelli G, Cotroneo CE, Noci S, Dugo M, Galvan A, Delli Carpini S, et al. Genetic susceptibility variants for lung cancer: replication study and assessment as expression quantitative trait loci. Sci Rep. 2017 Feb 9;7:42185. doi: 10.1038/srep42185 28181565 PMC5299838

[31] Zhou W, Liu G, Park S, Wang Z, Wain JC, Lynch TJ, et al, Christiani DC. Gene-smoking interaction associations for the ERCC1 polymorphisms in the risk of lung cancer. Cancer Epidemiol Biomarkers Prev. 2005 Feb;14(2):491–6. 15734977 10.1158/1055-9965.EPI-04-0612

[32] Gao H, Ge RC, Liu HY, Wang Y, Yan S. Effect of ERCC1 polymorphism on the response to chemotherapy and clinical outcome of non-small cell lung cancer. Genet Mol Res. 2014 Oct 31;13(4):8997–9004. doi: 10.4238/2014.October.31.14 25366790

[33] Isla D, Sarries C, Rosell R, Alonso G, Domine M, Taron M, et al. Single nucleotide polymorphisms and outcome in docetaxel-cisplatin-treated advanced non-small-cell lung cancer. Ann Oncol. 2004 Aug;15(8):1194–203. doi: 10.1093/annonc/mdh319 15277258

[34] Pérez-Ramírez C, Cañadas-Garre M, Alnatsha A, Villar E, Valdivia-Bautista J, Faus-Dáder MJ, et al. Pharmacogenetics of platinum-based chemotherapy: impact of DNA repair and folate metabolism gene polymorphisms on prognosis of non-small cell lung cancer patients. Pharmacogenomics J. 2019 Apr;19(2):164–177. doi: 10.1038/s41397-018-0014-8 29662106

[35] Takenaka T, Yano T, Kiyohara C, Miura N, Kouso H, Ohba T, et al. Effects of excision repair cross-complementation group 1 (ERCC1) single nucleotide polymorphisms on the prognosis of non-small cell lung cancer patients. Lung Cancer. 2010 Jan;67(1):101–7. doi: 10.1016/j.lungcan.2009.03.007 19361884

[36] Yu T, Xue P, Cui S, Zhang L, Zhang G, Xiao M, et al. Rs3212986 polymorphism, a possible biomarker to predict smoking-related lung cancer, alters DNA repair capacity via regulating ERCC1 expression. Cancer Med. 2018 Dec;7(12):6317–6330. doi: 10.1002/cam4.1842 30453383 PMC6308093

[37] Li D, Shi J, Liang D, Ren M, He Y. Lung cancer risk and exposure to air pollution: a multicenter North China case-control study involving 14604 subjects. BMC Pulm Med. 2023 May 24;23(1):182. doi: 10.1186/s12890-023-02480-x 37226220 PMC10210434

[38] Gudmundsson G, Tomasson K. Asbestos and its effects on health of Icelanders—review. Laeknabladid. 2019 Juli;105(7):327–334. doi: 10.17992/lbl.2019.0708.241 31411568

[39] Ge C, Peters S, Olsson A, Portengen L, Schüz J, Almansa J, et al. Respirable Crystalline Silica Exposure, Smoking, and Lung Cancer Subtype Risks. A Pooled Analysis of Case-Control Studies. Am J Respir Crit Care Med. 2020 Aug 1;202(3):412–421. doi: 10.1164/rccm.201910-1926OC 32330394 PMC7465090

[40] Manno M, Levy L, Johanson G, Cocco P. Silica, silicosis and lung cancer: what level of exposure is acceptable? Med Lav. 2018 Dec 20;109(6):478–480. doi: 10.23749/mdl.v109i6.7928 30556538 PMC7682183

[41] Rayens NT, Rayens EA, Tighe RM. Co-occurrence of pneumoconiosis with COPD, pneumonia and lung cancer. Occup Med (Lond). 2022 Dec 7;72(8):527–533. doi: 10.1093/occmed/kqac079 35932472

[42] Tomášková H, Horáček J, Šlachtová H, Šplíchalová A, Riedlová P, Dalecká A, et al. Analysis of Histopathological Findings of Lung Carcinoma in Czech Black Coal Miners in Association with Coal Workers’ Pneumoconiosis. Int J Environ Res Public Health. 2022 Jan 9;19(2):710. doi: 10.3390/ijerph19020710 35055532 PMC8775382

[43] Patel J, Baptiste BA, Kim E, Hussain M, Croteau DL, Bohr VA. DNA damage and mitochondria in cancer and aging. Carcinogenesis. 2020 Dec 31;41(12):1625–1634. doi: 10.1093/carcin/bgaa114 33146705 PMC7791626

[44] Poli G, Schaur RJ, Siems WG, Leonarduzzi G. 4-hydroxynonenal: a membrane lipid oxidation product of medicinal interest. Med Res Rev. 2008 Jul;28(4):569–631. doi: 10.1002/med.20117 18058921

[45] Feng Z, Hu W, Tang MS. Trans-4-hydroxy-2-nonenal inhibits nucleotide excision repair in human cells: a possible mechanism for lipid peroxidation-induced carcinogenesis. Proc Natl Acad Sci U S A. 2004 Jun 8;101(23):8598–602. doi: 10.1073/pnas.0402794101 15187227 PMC423240

[46] Winczura A, Czubaty A, Winczura K, Masłowska K, Nałęcz M, Dudzińska DA, et al. Lipid peroxidation product 4-hydroxy-2-nonenal modulates base excision repair in human cells. DNA Repair (Amst). 2014 Oct;22:1–11. doi: 10.1016/j.dnarep.2014.06.002 25083554

[47] Czerwińska J, Nowak M, Wojtczak P, Dziuban-Lech D, Cieśla JM, Kołata D, et al. ERCC1-deficient cells and mice are hypersensitive to lipid peroxidation. Free Radic Biol Med. 2018 Aug 20;124:79–96. doi: 10.1016/j.freeradbiomed.2018.05.088 29860127 PMC6098728

[48] Kim E, Ahuja A, Kim MY, Cho JY. DNA or Protein Methylation-Dependent Regulation of Activator Protein-1 Function. Cells. 2021 Feb 21;10(2):461. doi: 10.3390/cells10020461 33670008 PMC7926996

[49] Huerta Guevara AP, McGowan SJ, Kazantzis M, Stallons TR, Sano T, Mulder NL, et al. Increased insulin sensitivity and diminished pancreatic beta-cell function in DNA repair deficient Ercc1d/- mice. Metabolism. 2021 Apr;117:154711. doi: 10.1016/j.metabol.2021.154711 33493548 PMC8625516

[50] Park SS, Seo YK. Excess Accumulation of Lipid Impairs Insulin Sensitivity in Skeletal Muscle. Int J Mol Sci. 2020 Mar 12;21(6):1949. doi: 10.3390/ijms21061949 32178449 PMC7139950

[51] Fu CZ, Jia L, Wang DP et al (2017) Diagnostic value of serum osteopontin for pneumoconiosis patients combined with heart failure[J]. Occupational Health and Emergency Rescue, 2017, 35(4): 308–310, 355.

[52] Yan Z, Cheng X, Wang T, Hong X, Shao G, Fu C. Therapeutic potential for targeting Annexin A1 in fibrotic diseases. Genes Dis. 2022 Jun 18;9(6): 1493–1505. doi: 10.1016/j.gendis.2022.05.038 36157506 PMC9485289

[53] Lin W, Chen H, Chen X, Guo C. The Roles of Neutrophil-Derived Myeloperoxidase (MPO) in Diseases: The New Progress. Antioxidants (Basel). 2024 Jan 22;13(1):132. doi: 10.3390/antiox13010132 38275657 PMC10812636

[54] Melchionna R, Trono P, Tocci A, Nisticò P. Actin Cytoskeleton and Regulation of TGFβ Signaling: Exploring Their Links. Biomolecules. 2021 Feb 23;11(2):336. 33672325 10.3390/biom11020336 PMC7926735

[55] Zhao W, Wang L, Wang Y, Yuan H, Zhao M, Lian H, et al. Injured Endothelial Cell: A Risk Factor for Pulmonary Fibrosis. Int J Mol Sci. 2023 May 14;24(10):8749. doi: 10.3390/ijms24108749 37240093 PMC10218114

